# Transcription factor 4 is a key mediator of oncogenesis in neuroblastoma by promoting MYC activity

**DOI:** 10.1002/1878-0261.13714

**Published:** 2024-08-09

**Authors:** Nour A. Aljouda, Dewan Shrestha, Chelsea DeVaux, Rachelle R. Olsen, Satyanarayana Alleboina, Megan Walker, Yong Cheng, Kevin W. Freeman

**Affiliations:** ^1^ Department of Genetics, Genomics and Informatics University of Tennessee Health Science Center Memphis TN USA; ^2^ Department of Hematology St. Jude Children's Research Hospital Memphis TN USA; ^3^ Department of Oncological Sciences Huntsman Cancer Institute Salt Lake City UT USA

**Keywords:** core regulatory circuit, MYCN, neuroblastoma, super‐enhancer, TCF4, transcription factor

## Abstract

Super‐enhancer‐associated transcription factor networks define cell identity in neuroblastoma (NB). Dysregulation of these transcription factors contributes to the initiation and maintenance of NB by enforcing early developmental identity states. We report that the class I basic helix–loop–helix (bHLH) transcription factor 4 (*TCF4*; also known as *E2‐2*) is a critical NB dependency gene that significantly contributes to these identity states through heterodimerization with cell‐identity‐specific bHLH transcription factors. Knockdown of *TCF4* significantly induces apoptosis *in vitro* and inhibits tumorigenicity *in vivo*. We used genome‐wide expression profiling, TCF4 chromatin immunoprecipitation sequencing (ChIP‐seq) and TCF4 immunoprecipitation–mass spectrometry to determine the role of TCF4 in NB cells. Our results, along with recent findings in NB for the transcription factors T‐box transcription factor TBX2, heart‐ and neural crest derivatives‐expressed protein 2 (HAND2) and twist‐related protein 1 (TWIST1), propose a role for TCF4 in regulating forkhead box protein M1 (FOXM1)/transcription factor E2F‐driven gene regulatory networks that control cell cycle progression in cooperation with N‐myc proto‐oncogene protein (MYCN), TBX2, and the TCF4 dimerization partners HAND2 and TWIST1. Collectively, we showed that TCF4 promotes cell proliferation through direct transcriptional regulation of the c‐MYC/MYCN oncogenic program that drives high‐risk NB. Mechanistically, our data suggest the novel finding that TCF4 acts to support MYC activity by recruiting multiple factors known to regulate MYC function to sites of colocalization between critical NB transcription factors, TCF4 and MYC oncoproteins. Many of the TCF4‐recruited factors are druggable, giving insight into potential therapies for high‐risk NB. This study identifies a new function for class I bHLH transcription factors (e.g., TCF3, TCF4, and TCF12) that are important in cancer and development.

## Graphical abstract

Core regulatory circuitry (CRC) transcription factors (TFs), whose effects are increased by MYC, define cancer cell‐type identity and drive oncogenes in cancer. This study found that the TF TCF4 (E2‐2) heterodimerizes with key CRC TFs to recruit MYC cofactors that increase MYC DNA‐binding and activity. These findings better define the mechanistic relationship of CRC TFs with MYC.

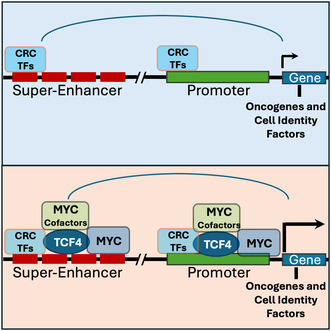

**Abbreviations**
ADRN: adrenergic
BET proteins: bromodomain and extra‐terminal domain
bHLH: basic helix–loop–helix
CoREST: corepressor for element‐1‐silencing transcription factor
CRC: core regulatory circuits
Dox: doxycycline
EMT: epithelial to mesenchymal transition
GSEA: Gene Set Enrichment Analysis
HAND2: neural crest derivatives‐expressed protein 2
HDAC: histone deacetylases
KD: knockdown
MES: mesenchymal
MYCN: N‐myc proto‐oncogene protein
NB: neuroblastoma
NCCs: neural crest cells
SEdb: *in‐silico* approach utilizing the super‐enhancer database
SEs: super‐enhancers
SNS: sympathetic nervous system
TBX2: T‐box transcription factor
TCF4: transcription factor 4
TFs: transcription factors
TWIST1: twist‐related protein 1

## 1 Introduction

Neuroblastomas (NBs) are aggressive pediatric tumors that originate from the sympathoadrenal lineage of neural crest cells (NCCs) and account for 15% of all pediatric cancer deaths [1]. Patients with MYCN amplification are categorized as high‐risk NB due to the aggressive nature of these cancers. Non‐MYCN‐amplified high‐risk NB is often driven by overexpression of *c‐MYC* [2]. NB arises from a developmental block in NCC differentiation along the sympathoadrenal nervous system lineage leading to uncontrolled cell growth [3, 4]. The transition between identity states in differentiation is governed by different sets of master transcription factors (TFs) driven by super‐enhancers (SEs) [5, 6]. These factors autoregulate their own gene expression and collectively form core regulatory circuits (CRC) that drive high expression of downstream lineage‐specific genes [7]. Two undifferentiated states exist in NB tumors: a more proliferative early adrenergic (ADRN) cell state and a more immature invasive mesenchymal (MES) cell state [8, 9]. Cells have the capacity to shift between these two states, providing these tumors with remarkable transcriptional plasticity and the ability to escape therapy [9, 10].

Bromodomain and extra‐terminal domain (BET) proteins are epigenetic readers that promote transcription and are enriched at SEs, large genomic clusters occupied by high levels of TFs, and BET‐dependent regulatory regions [11]. NB is particularly sensitive to BET inhibitors (BETi); however, the reasons for this are not fully understood [12, 13]. BET proteins, such as BRD4, bind acetylated lysine at histones and stabilize transcriptional complexes. Importantly, SEs drive the expression of both oncogenes (e.g., N‐Myc) and regulators of cell identity [12]. In NB N‐MYC typically binds canonical E‐Boxes at promoters, but at oncogenic levels of expression *N‐MYC* invades and binds to noncanonical E‐boxes at SEs [14]. Both the ADRN and MES CRCs are reliant on class II bHLH TFs such as *HAND2 and TWIST1* [15]. Both HAND2 and TWIST1 were shown to bind near N‐MYC binding sites at N‐MYC invaded SEs, facilitate N‐MYC DNA binding and were necessary for N‐MYC driven proliferation [14, 16]. N‐MYC enhancer invasion in NB recapitulates the c‐MYC enhancer invasion observed in cancers with elevated expression of c‐MYC [17]. Though HAND2 and TWIST1 improve the DNA binding of N‐Myc how they achieve this is poorly understood.

Using BETi as a tool and interrogation of SEs databases, we identified the E‐box transcription factor *TCF4* as a promising candidate for regulating both the ADRN and MES cell identities. *TCF4* is a class I basic helix–loop–helix (bHLH) TF that heterodimerizes with several class II bHLH factors known to regulate tissue development, cell differentiation, and pathological disease [18, 19]. *TCF4* is a dimerization partner of TWIST1, a critical determinant of mesenchymal development [19]. Furthermore, TCF4 heterodimerizes with proneuronal TFs, such as Achaete‐scute homolog 1 (ASCL1), facilitating their ability to regulate the differentiation of neuronal progenitors [18]. *TCF4* is essential for epithelial to mesenchymal transition (EMT) [20], human nervous system development [21], B‐ and T‐cell development [22, 23], and the regulation of neural stem cell (NSC) identity [24]. In cancer, *TCF4* is a master transcriptional regulator that sustains malignancy in the blastic plasmacytoid dendritic cell neoplasm (BPDCN) [25], it contributes to aggressive bone colonization in human lung carcinoma cell lines [26], and high *TCF4* expression is associated with worse outcomes in acute myeloid leukemia [27]. However, how E‐box transcription factors, like TCF4, functionally contribute to development and malignancy is poorly characterized.

Here, we confirmed that TCF4 interacts with multiple CRC TFs, and we report that *TCF4* is a lineage‐dependency factor in both ADRN and MES NB cells. A comparative analysis of TCF4 and multiple CRC factors identified TCF4 as a critical part of the extended regulator network reported to work downstream of CRC TFs. Our findings reveal a previously unknown role of TCF4 in recruiting factors that enhance MYC activity to CRC heterodimerization partners and a novel understanding of TCF4's function in development and cancer.

## 2 Materials and methods

### 2.1 Cell lines

All neuroblastoma cell lines, Kelly (RRID: CVCL_2092) SK‐N‐AS (RRID: CVCL_1700), IMR32, and NIH3T3, were obtained from ATCC (Manassas, VA, USA). Primary mouse NCC lines were isolated and cultured as described previously [28, 29]. We isolated the neural tube of day 9.5 embryos from a region caudal to the heart to the most caudal somite. The isolated neural tubes are then cultured in chemically defined medium (CDM) for 48 h, to allow NCC migration onto the plate. contains Iscove's modified Dulbecco's medium/Ham's F‐12 1τ1, 1× chemically defined lipid concentrate (GIBCO, Grand Island, NY, USA), 1× μg·mL^−1^ Insulin‐transferrin‐selenium (Thermo Fisher Scientific, Waltham, MA, USA), 450 μm monothioglycerol (Sigma, Burlington, MA, USA), 5 mg·mL^−1^ purified BSA (Sigma), 7 μg·mL^−1^ Insulin (Thermo Fisher Scientific), and penicillin/streptomycin (Invitrogen, Carlsbad, CA, USA). Culture dishes were coated with fibronectin (250 μg·mL^−1^) (Corning, Corning, NY, USA). The medium was supplemented with EGF (R&D, Minneapolis, MN, USA) and FGF2 (R&D) to modulate growth factor signals and SB431542 (SB) (Sigma) as a TGF‐beta signaling pathway suppressor.

Cell lines were tested against Mycoplasma using LookOut Mycoplasma PCR Detection Kit (Sigma‐Aldrich, St. Louis, MO, USA). Cell line authentication was performed using short tandem repeat (STR) genotyping.

### 2.2 CyQuant assay

Ten thousand cells/well were plated in each well of a 96‐well plate, 24‐h later drug was added, the plates were incubated for 4 days and then subjected to a CyQuant Cell Direct Proliferation Assay (Thermo Fisher Scientific) and read using a Cytation 5 plate reader (BioTek, Winooski, VT, USA).

### 2.3 Real‐time quantitative PCR

Total RNA was extracted using the RNeasy Mini Kit (QIAGEN, Germantown, MD, USA). cDNA synthesis was performed using the High‐Capacity cDNA Reverse Transcription Kit (Thermo Fisher Scientific). Real‐time quantitative polymerase chain reaction (qPCR) was performed using TaqMan Real‐Time PCR Master Mix. The housekeeping gene *B2M* was used for normalization. The following human probes were used: Hs00972432_m1 for TCF4 and Hs00187842_m1 for B2M, and mouse probes: Mm00443210_m1 for tcf4 and Mm00437762_m1 for b2m.

### 2.4 Co‐immunoprecipitation and western blotting analysis

Total protein was extracted from Kelly and SK‐N‐AS cells using Pierce IP lysis buffer (#87787; Thermo Fisher Scientific) supplemented with Halt Protease and Phosphatase Inhibitor Cocktail (#1861281). Whole‐cell lysates were incubated with TCF4 antibody (ab217668; Abcam, Burlington, CA, USA) or Normal Rabbit IgG (#2729; CST, Danvers, MA, USA) overnight at 4 °C. Lysate was then mixed with prewashed Pierce Protein A/G Magnetic Beads (#88803; Thermo Fisher Scientific) and incubated. Next, beads were separated from the lysate using a magnetic separation rack, washed with IP lysis buffer, and eluted with 4× SDS sample buffer and boiled. For input, 10% of cell lysate was used. Proteins were separated by sodium dodecyl sulfate/polyacrylamide gel electrophoresis (SDS/PAGE). The following antibodies were used to detect the protein–protein interactions: TCF4 (ab217668; Abcam), ASCL1 (#sc‐390794; Santa Cruz, Dallas, TX, USA), HAND2 (ab200040; Abcam), and TWIST1 (ab50887; Abcam).

### 2.5 Doxycycline‐inducible shRNA systems

The shRNA sequences were designed according to the TRC1 library (Sigma‐Aldrich, TRCN0000274214, TRCN0000274213, and TRCN0000274161, referred to in the manuscript as sh1, sh2, and sh3, respectively) targeting TCF4. The RNAs were cloned into the lentiviral vector Tet‐pLKO‐puro (Plasmid #21915) and lentivirus for each shRNA was generated with 293 T cells using second‐generation lentiviral system. Kelly and SK‐N‐AS cells were infected with the lentivirus in the presence of polybrene (4 μg·mL^−1^; Millipore, Burlington, MA, USA). Selection was performed by adding puromycin 48 h after infection.

### 2.6 Colony formation assays

50 000 NB cells were seeded in a 6‐cm dish. Cells were incubated for 10 days in media only or doxycycline, with medium changes every 2 days. Colonies were stained with 0.05% crystal violet.

### 2.7 Annexin V/PI flow cytometry assay

Kelly and SK‐N‐AS cells transduced with shRNA against TCF4 (sh1, sh2, and sh3) were treated with 1 μg·mL^−1^ doxycycline for 5 and 7 days, respectively. Cells were then analyzed by flow cytometry using the Annexin V‐FITC/PI detection kit obtained from BioLegend, San Diego, CA, USA (#640914). Representative data from three biological replicates are presented.

### 2.8 Ectopic expression of *TCF4*

The TCF4/E2‐2 cDNA ORF Clone (#HG12096‐CF; Sino Biologica, Houston, TX, USA) was transfected into SK‐N‐AS cells using FuGENE 6 transfection reagent (Roche, Indianapolis, IN, USA). Hygromycin‐resistant clones were selected and expanded.

### 2.9 Colony formation in soft agar

NB cells stably transduced with TCF4 shRNA were seeded (5000 cells/well) in 0.30% noble agar (Sigma) mixed with the culture medium (topping 0.6% noble agar with medium). Cells were incubated in doxycycline or medium alone for 21 days.

### 2.10 *In vivo* tumor models

This study was performed in strict accordance with the recommendations of the Guide for the Care and Use of Laboratory Animals of the National Institutes of Health. All animal experiments were approved by the Institutional Animal Care and Use Committee (IACUC‐#22‐0409) at UTHSC. Animal facilities are maintained on a 12 : 12 h light–dark cycle at 20–23 °C and 40–50% relative humidity. Female 6–8‐week‐old SCID (IcrTac:ICR‐*Prkdc* ^*scid*^) mice were obtained from (Taconic Biosciences, Germantown, NY, USA) and Female SCID mice were subcutaneously flank injected with 2 × 10^6^ cells per mouse. Kelly and SK‐N‐AS cells transduced with shRNA against TCF4 (Sh2) were injected in a 1 : 1 mix of cells and Matrigel (BD Biosciences, Franklin Lakes, NJ, USA). One week after the injection, the mice were randomly assigned to the control (standard diet) or treatment (15 mg·kg^−1^ body weight (BW) of doxycycline) groups. Tumors were caliper measured twice‐weekly and tumor volume was calculated using the formula 1/2 × (length × width^2^). Animals were sacrificed according to institutional guidelines when the tumors reached approximately 2000 mm in length or width.

### 2.11 RNA‐seq library preparation and sequencing

The total RNA of Kelly and SK‐N‐AS human neuroblastoma cell lines were extracted using RNeasy Mini Kit (QIAGEN). cDNA libraries were sequenced using the Illumina NovaSeq platform (Novogene, Sacramento, CA, USA). star (version 2.5.3a) [30] was used to map sequencing data to the human reference genome (hg38). mGene abundance count was calculated using featurecounts (Subread version 1.5.1) [31]. The parameter “‐p” was used for featurecounts instead of reads to obtain fragment‐based counts. gencode v41 (hg38) was used to quantify gene expression. deseq2 [12, 32] from the r package was used to identify differentially expressed genes. Genes with low coverage were removed if their median value was < 0. Batch information, along with the treatment conditions were used as the design matrix. Genes differentially expressed after the knockdown of *TCF4* were selected using the following criteria: adjusted *P*‐value <  0.05, log 2‐fold change < −0.58 or > 0.58. deseq2 normalized data were used as input for GSEA (version 4.2.2) [33] for gene ontology analysis against the hallmark database (version 7.5.1).

### 2.12 Chromatin immunoprecipitation ChIP‐seq

A total of 3 × 10^7^ cells were cross‐linked with 1% formaldehyde (Sigma), quenched using 125 mm glycine (#G8898; Sigma) and DNA was sheared by sonication to approximately 500 bp. Antibodies anti‐TCF4 (ab217668; Abcam) or anti‐H3K27ac (ab4729) were prebound to protein A/G agarose beads (#20423; Thermo Fisher). Chromatin fragments were immunoprecipitated with total chromatin samples processed in parallel as input references. After reverse cross‐linking DNA purification was performed using the Qiagen MinElute PCR Purification Kit (#28004). TCF4 ChIP‐seq was done on an Illumina PE150 sequencer (Novogene). bwa‐mem (version 0.7.16a) [34] with default parameters was used to map the sequence data to the human genome (hg38). macs2 (version 2.2.1) [35] was used to identify the peaks. For the TCF4 ChIP‐seq data, idr (version 2.0.3) peaks were called at an IDR threshold of 0.05. Homer motif analysis was performed using IDR peaks generated from TCF4 ChIP‐seq data.

### 2.13 Target genes

TCF4 ChIP‐seq data, along with promoter capture Hi‐C data, were integrated with RNA‐seq data to identify target genes. Only differentially expressed genes with a fold‐change threshold of 1.5 and a *P*‐value < 0.05 were used for this analysis. If the promoter region or gene body overlaps with the coordinates of the TCF4 peak region, or if, based on promoter capture Hi‐C data, the region overlapping with TCF4 peaks interacts with the genes, those genes are defined as the target genes. bwa‐mem (version 0.7.16a) [34] with default parameters was used to map sequence data to the human genome (hg38). samtools (version 1.9) was used to remove the duplicate reads from the mapped file. CPM normalization was used to generate the signal track (bigiwig file) using bamcoverage (version 3.5). Adrenal gland‐associated promoter capture Hi‐C interaction data were downloaded from a study by Jung et al. [36]. Significant interactions between promoter‐other and promoter‐promoter based on TCF4 ChIP Seq and Promoter Capture Hi‐C were used. Promoters were defined 1000 bp upstream of the transcription start site (hg38 gencode v41) based on the strand information. Enhancers for the respective cell lines were defined based on the overlap between H3K27ac and H3K4me1 peaks using bedtools intersect [37].

### 2.14 Public data

ATAC Seq data (SRA: SRR10215668, SRR10215669) and H3K4me1 data (SRA: SRR10217411, SRR10217413) for Kelly were downloaded from NCBI GEO [38]. ChIP‐seq data for GATA3, ISL1, HAND2, n‐Myc, PHOX2B, and TBX2 data for Kelly were downloaded from NCBI GEO [8]. ATAC Seq data (SRA: SRR10215682, SRR10215683), H3k4me1, and HK3K27ac (SRA: SRR10217389, SRR10217391, SRR10217393) data for SKNAS were downloaded from NCBI GEO [38].

### 2.15 Immunoprecipitation‐mass spectrometry analysis

Immunoprecipitation of TCF4 in Kelly and SK‐N‐AS cell lines was performed as described in the Co‐immunoprecipitation method section. The IP‐MS protocol was performed in triplicate in parallel with an IgG control. Antibody: Bead complex samples were sent to the University of Tennessee Health Science Center Proteomics Core for Label‐Free Quantification (LFQ) mass spectrometry for protein identification. After trypsin digestion peptides were analyzed using Proteome Discoverer 2.4, (Thermo Fisher, Waltham, MA, USA). Peptide Abundance represents the MS Peak Area, the normalization mode is the total peptide amount, and protein abundance is the summed abundance of the assigned peptides. TCF4 interaction partners were included in the final list if they were present in at least two out of three TCF4 complex purifications and showed at least two‐fold enrichment by protein abundance in the TCF4 purified sample compared to the control sample. Cytoskeletal and cytoplasmic proteins (UniProt) were removed.

### 2.16 Statistical analysis

The statistical analyses used throughout this paper are specified in the appropriate results paragraphs and Section 2. We used the unpaired two‐tailed Student's *t*‐test to determine statistical significance using the graphpad prism software (Boston, MA, USA).

## 3 Results

### 3.1 Neuroblastoma sensitivity to BET inhibition is due to transcriptional addiction, not oncogene addiction

**Fig. 1:**
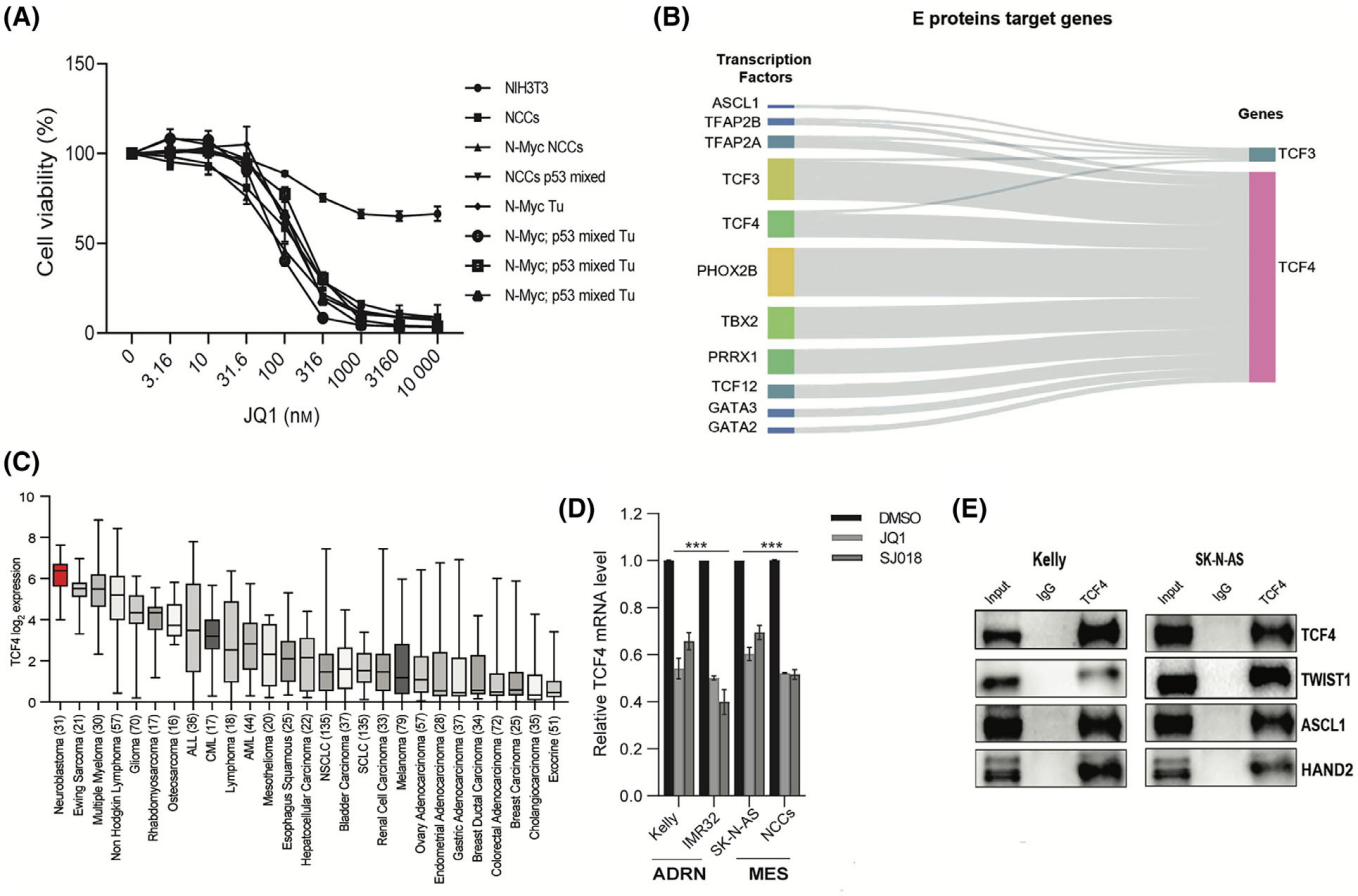
TCF4 is highly expressed in neuroblastoma and JQ1 suppresses the expression of TCF4 in multiple neuroblastoma lineage cells. (A) Dose–response of cells after 4‐day treatment with half‐log dilutions of the BET inhibitor JQ1. Cell lines include neural crest cells (NCCs), NCCs overexpressing N‐Myc (N‐Myc NCC), NCCs pooled from p53−/− and p53+/− embryos (NCC p53 mixed), cell line from N‐Myc tumor (N‐Myc Tu), cell lines from N‐Myc p53 mixed tumors (N‐Myc; p53 mixed Tu) and control NIH3T3. Results were normalized to control + SE. *n* = 3 independent experiments. (B) A relationship plot generated from the Super‐enhancer database (SEdb 2.0) using the SE‐based transcription factor (TF) TF‐GENE Analysis program to comprehensively analyze TFs gene pairs mediated by super‐enhancers (SEs) in neuroblastoma (NB) cell lines. (C) TCF4 expression (log_2_) in NB tumors and NB cell lines as compared to other tumors or cell lines. Boxplots show the 1st quartile up to the 3rd quartile of the data values and median as a line within the box. Number of samples for each cancer are shown in parenthesis. (D) TCF4 mRNA levels were determined by quantitative real‐time PCR after NB cell lines and primary NCCs were treated with DMSO, 1 μm JQ1 or SJ018 for 3 h. Expression values are shown relative to the DMSO condition for each cell line. *n* = 3 biological replicates. Data are presented as the mean ± SE two tailed unpaired *t*‐test (***P < 0.001 vs. control). (E) Immunoprecipitation (IP) of TCF4 using Kelly and SK‐N‐AS whole lysate. *n* = 3 independent experiments, and the clearest mages from these replicates were selected for presentation. Western blot is probed with the indicated antibodies. Control IP by rabbit IgG and 10% input are also shown.

Targeting epigenetic regulators such as BET proteins (e.g., BRD4) has emerged as a powerful therapeutic strategy for cancer treatment [12]. NB is one of the most BETi‐sensitive cancers, which was attributed to MYCN amplification, an oncogenic driver found in approximately 20% of high‐risk NB. However, ectopic *MYCN* expression in NB cell lines did not rescue cells from BETi [13]. To better understand NB response to BET bromodomain inhibition, we transformed primary murine NCCs, the NB cell of origin, into NB by enforcing *N‐Myc* expression [29]. Here, we generated a panel of cell lines at different stages of NB transformation, including normal NCCs, NCCs with *N‐Myc* overexpression, p53−/− and p53+/− mixed NCCs (p53mixed NCCs), and tumor‐derived cell lines (TDCL) from an NB tumor caused by *N‐Myc* overexpression in wild‐type NCCs (N‐Myc Tu), and TDCLs from three independent tumors caused by *N‐Myc* overexpression in p53mixed NCCs (N‐Myc; p53mixed Tu) [29]. Based on the two current models for BETi cytotoxicity, an oncogene addiction model would predict that primary NCCs would be resistant to BETi, while a lineage sensitivity model, also characterized as transcriptional addiction [39], would predict that all NCC‐derived lines would be BETi‐sensitive. Supporting the lineage sensitivity model, these cell lines displayed equivalent sensitivity to JQ1 treatment (Fig. 1A). NIH3T3 cells were relatively unaffected, indicating that JQ1 is not broadly toxic. NCCs were also more sensitive to JQ1 treatment than the human NB cell lines (Fig. S1a). These data suggest that the shared susceptibility of NCC‐derived lines to JQ1 is lineage‐dependent.

### 3.2 Neuroblastoma core regulatory circuits share TCF4

Recent studies have reported intratumoral heterogeneity within NB tumors, which plays a major role in resistance to therapy. These heterogeneous tumors include a majority of committed ADRN tumor cells and a minority of MES/NCC‐like cells [10]. Based on finding lineage‐dependency in NB, we set out to identify a novel master TF whose expression is sensitive to BETi, found across neuroblastomas, and shared by both the MES and ADRN NB subtypes. Given that master TFs that control cell identity in NB can be defined by super‐enhancers, we used an *in‐silico* approach utilizing the super‐enhancer database (SEdb) to identify super‐enhancer regulated TFs that are broadly regulated by NCC and NB TFs [40]. Here, we interrogated a comprehensive list of TFs that specify multiple stages of NCC development toward a commitment to early sympathoadrenal neurons including ADRN CRC factors, MES CRC factors, and transcription factor AP‐2 (TFAP2) [41]. We included the class I bHLH TFs (TCF3, TCF4, and TCF12) that heterodimerize with many of the CRC TFs. We tested early developmental sympathetic nervous system (SNS) TFs against the SEs of the class I bHLH TFs (Fig. 1B), ADNR TFs (Fig. S1c) and MES TFs (Fig. S1c). SEdb SE‐based TF‐GENE analysis program predicted TCF4 to be regulated by TFs that are critical for controlling early stages of SNS development including the master regulator of early SNS commitment paired‐like homeobox 2B gene (*PHOX2B*), a core MES TFs paired related homeobox 1 (*PRRX1*), pioneer TFs gata binding proteins (*GATA2* and 3), and *TFAP2* TFs, which are CRC independent but are important early SNS factors (Fig. 1B and Fig. S1c). Moreover, we found that *TCF4* has the highest expression in NB of all cancer types found in the Cancer Cell line Encyclopedia (CCLE) database (Fig. 1C). Next, we determined that JQ1 and SJ018, a structurally distinct BETi, suppress *TCF4* expression significantly by quantitative PCR (qRT‐PCR) in the human Kelly and IMR‐32 NB cell lines (ADRN) and the heterogeneous SK‐N‐AS cell line that skews MES, as well as primary mouse NCCs (Fig. 1D). TCF4 has been shown to interact with ASCL1 and TWIST1 [42, 43]. Using the STRING database [44], we found that TCF4 is highly connected by putative protein–protein interactions with many ADRN and MES CRC factors (Fig. S1b). To validate TCF4 interactions in NB, we purified endogenous TCF4 by immunoprecipitation (IP) and confirmed using IP‐WB that TCF4 interacts with ASCL1, HAND2, and TWIST1 in NB cell lines (Fig. 1E). Our findings identified TCF4 as a promising shared TF regulated by SEs across both identity states that itself could potentially regulate these identity states.

### 3.3 *TCF4* is essential for NB viability and a critical determinant of JQ1 sensitivity in NB cells

**Fig. 2:**
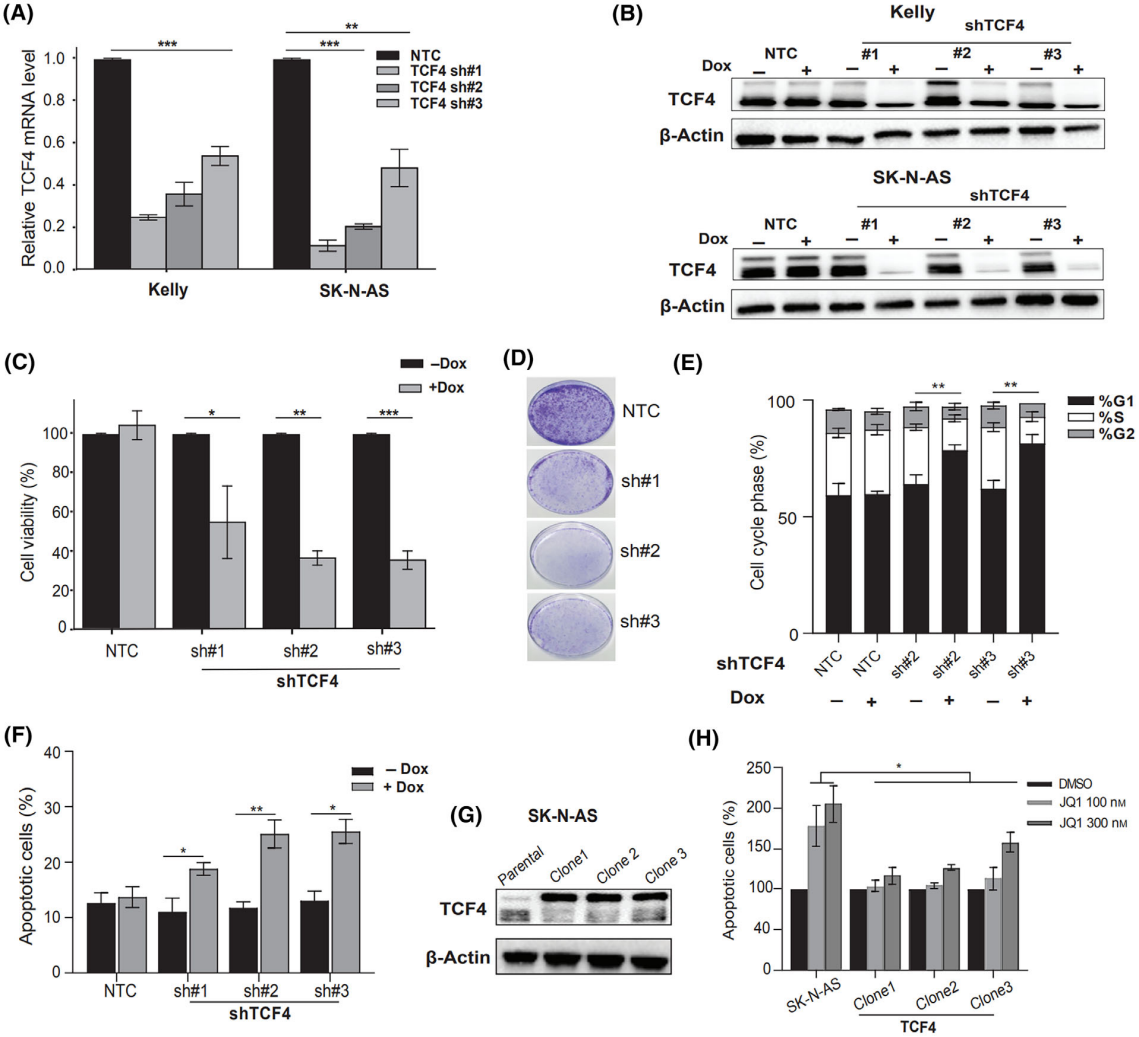
TCF4 is essential for neuroblastoma viability. (A) Quantitative RT‐PCR analysis showing TCF4 expression in Kelly and SK‐N‐AS stable cells treated with or without 1 μg·mL^−1^ doxycycline for 3 days. Data are presented as the means ± SE from three independent experiments. Two tailed unpaired *t*‐test (**P < 0.01, ***P < 0.001 vs. control). (B) Whole‐cell protein lysates were analyzed by western blotting using TCF4 antibody 5 days after 1 μg·mL^−1^ doxycycline treatment. *N* = 3 independent experiments, and the clearest mages from these replicates were selected for presentation. (C) CyQuant proliferation assay performed using SK‐N‐AS TCF4 sh #1, #2, #3 stable cell lines compared to empty vector control (NTC) cell line 7 days after doxycycline treatment. *n* = 3 biological replicates. Two tailed unpaired *t*‐test (*P < 0.05, **P < 0.01, ***P < 0.001 vs. control). (D) Colony formation assays were performed following TCF4 knockdown in SK‐N‐AS. Cells were cultured for 10 days in the presence or absence of 1 μg·mL^−1^ of doxycycline. (E) % of cells in each phase of the cell cycle 5 days following TCF4 knockdown in the SK‐N‐AS cell line. Cell cycle was assayed by flow cytometry. *n* = 3 biological replicates. Data are presented as the mean ± SE two tailed unpaired *t*‐test (**P < 0.01 vs. control). (F) Quantitative analysis of the percentage of apoptotic cells (Annexin V+/FITC+) in SK‐N‐AS TCF4 stable cell lines treated with or without 1 μg·mL^−1^ doxycycline for 7 days. Two tailed unpaired *t*‐test (*P < 0.05, **P < 0.01, vs. control). (G) TCF4 overexpression was analyzed with western blot analysis in the stable SK‐N‐AS clones. (H) Annexin V/FITC staining of parental SK‐N‐AS cells and SK‐N‐AS overexpressing TCF4 cultured in the presence of increasing concentrations of the BET inhibitor JQ1 for 4 days. Data are presented as the means ± SE from three independent experiments. Two tailed unpaired *t*‐test (*P < 0.05 vs. control).

To determine the consequences of TCF4 knockdown (KD), we transduced two representative NB cell lines, Kelly (ADRN) and SK‐N‐AS (MES), with three doxycycline‐inducible shRNA expression vectors targeting *TCF4* (sh#1, sh#2, and sh#3). An empty vector harboring only (Tet‐pLKO‐puro) served as a control throughout the study. qRT‐PCR and western blot (WB) revealed that TCF4 was significantly downregulated in the established stable cell lines after treatment with doxycycline (Dox) (Fig. 2A,B). Loss of TCF4 in both cell lines led to a significant decrease in cell proliferation and colony formation ability compared with the control (Fig. 2C,D, and Fig. S2a,b). Moreover, we observed a significant G_1_ arrest and a decrease in S‐phase in both cell lines upon *TCF4* KD (Fig. 2E, Figs S2c and S3). Furthermore, TCF4 loss markedly induced apoptosis in both cell lines, as confirmed by Annexin V staining (Fig. 2F, Figs S2d and S4) and PARP cleavage, as detected by WB (Fig. S2e). Collectively, our data suggest that both NB identity states depend on TCF4 for growth and survival.

To interrogate TCF4‐dependent JQ1 effects, SK‐N‐AS cells were transfected with TCF4 cDNA clone. Three different clones overexpressing *TCF4* confirmed with qRT‐PCR (Fig. S2f) and WB analysis (Fig. 2G) were expanded and exposed to JQ1 for 4 days. JQ1‐induced apoptosis was blocked in cells ectopically expressing TCF4 at doses as high as 300 nm (Fig. 2H), indicating that *TCF4* is a sensitive and central component of JQ1 toxicity in NB.

### 3.4 TCF4 knockdown suppresses tumor growth *in vivo*

**Fig. 3:**
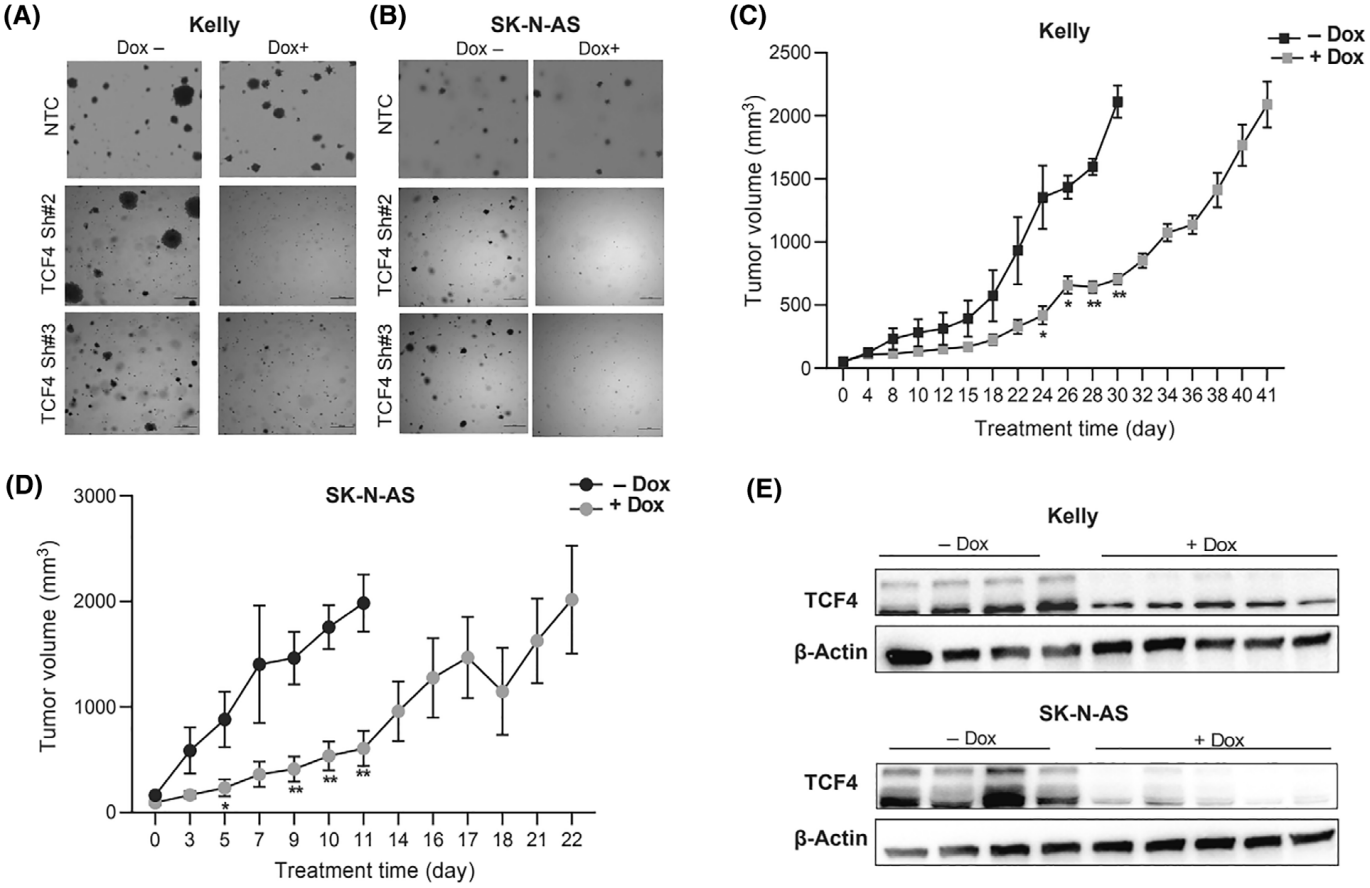
TCF4 knockdown suppresses tumor growth *in vivo*. (A, B) Soft‐agar colony formation assay (Clonogenic Assay) of the Kelly and SK‐N‐AS TCF4 stable cell lines at 21 days after doxycycline treatment. Experiments were performed in triplicates. Representative images are shown, scale bar, 500 μm. Growth curves for subcutaneous xenograft transduced with (C) Kelly TCF4 sh2, (D) SK‐NA‐S sh2 injected in the flank region of nude mice. One week after the injections, mice were assigned to either (−Dox) or (+Dox) feed (Con group = 4 mice, Dox group = 5 mice). Data is presented as the means ± SE two tailed unpaired *t*‐test (*P < 0.05, **P < 0.01). (E) TCF4 knockdown after doxycycline treatment was confirmed by immunoblot in tumors formed from Kelly and SK‐N‐AS neuroblastoma cells, control or transduced cells with an shRNA targeting TCF4. Experiments were performed in triplicates.

Next, the knockdown of *TCF4* in NB cells in a soft agar colony assay showed that colonies were smaller and significantly fewer in number for the *TCF4* KD cells compared to control, while doxycycline treatment itself did not affect the NTC cells lines (Fig. 3A,B). Next, we examined the effect of *TCF4* KD on NB subcutaneous xenograft tumors. Here, 2 × 10^6^ Kelly sh*TCF4* #2 and SK‐N‐AS sh*TCF4* #2 cells were implanted into the subcutaneous flank region of SCID mice separately. One week after the injection, the mice were randomized into two groups (+Dox and −Dox). We found that decreased expression of *TCF4* in Kelly and SK‐N‐AS cells delayed tumor growth *in vivo*, as reflected in the mean tumor volume over time compared with the control in both cell lines (Fig. 3C,D). The tumor tissues were subsequently analyzed by WB for TCF4 protein expression. As shown in Fig. 3E, Dox treatment markedly decreased *TCF4* expression in tumors whereas no significant change in TCF4 protein expression was observed in the absence of Dox in the control mice. Therefore, these results confirm our *in vitro* observations and further indicate that *TCF4* is a critical lineage‐dependency factor in NB.

### 3.5 TCF4 regulates genes critical for NB pathogenesis and cell identity states

**Fig. 4:**
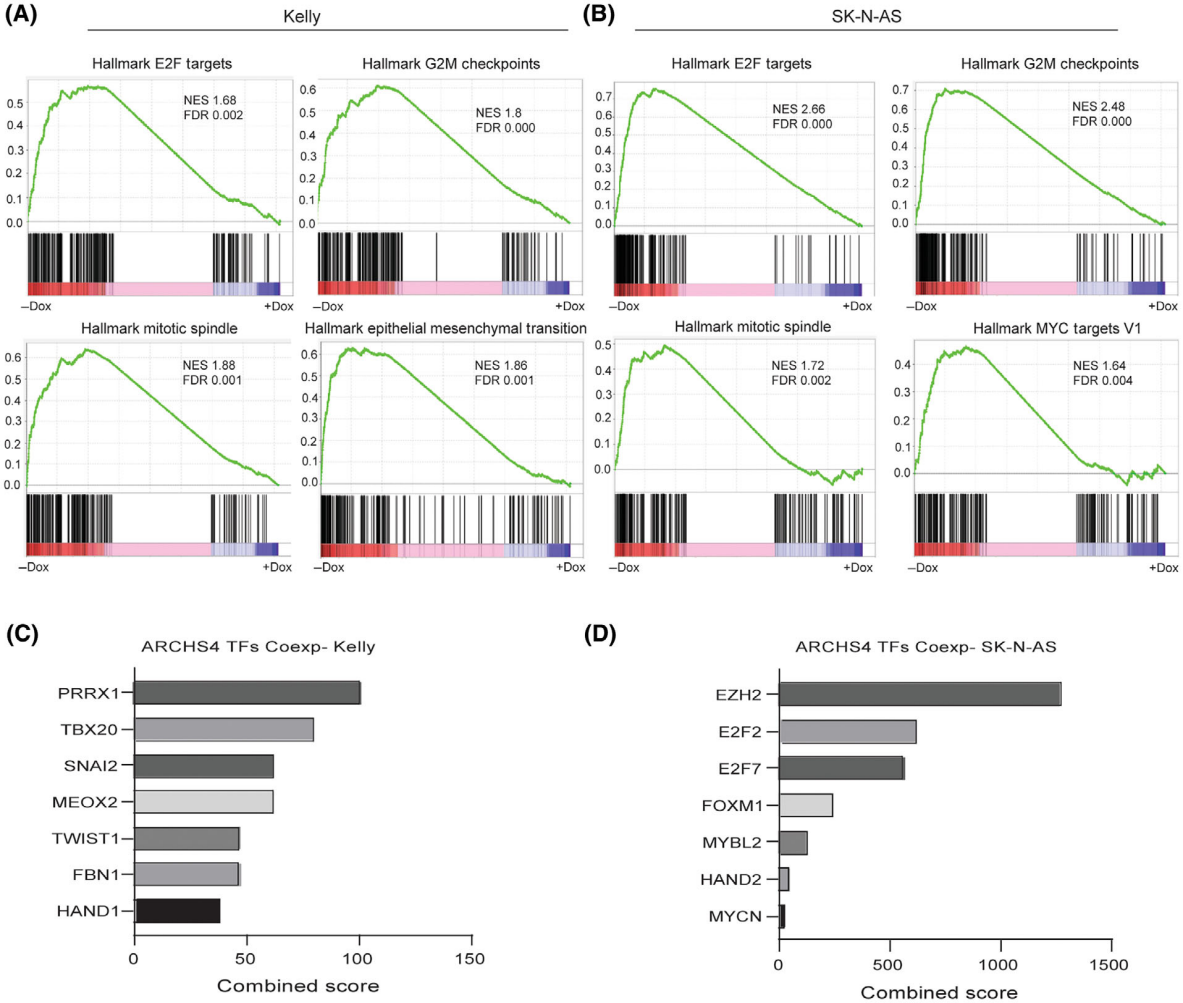
Knockdown of TCF4 deregulates gene expression of MYC target genes as well as genes involved in cell cycle. (A, B) Enrichment plots acquired from the gene set enrichment analysis (GSEA). Four significant pathways among the top enriched ones in vehicle‐treated cells compared to doxycycline‐treated cells upon TCF4 knockdown in Kelly and SK‐N‐AS. FDR < 0.01 was defined as statistically significant. (C, D) Enrichr pathway analysis of downregulated differentially expressed genes (DEG) following TCF4 knockdown in Kelly and SK‐N‐AS cells using the ARCHS4 TF Coexp. The lists of genes were analyzed based on the combined score ranking. *P*‐value < 0.05 was used as the significance threshold.

To investigate how TCF4 functions as an NB dependency gene, we performed RNA‐seq analysis following *TCF4 KD* in Kelly and SK‐N‐AS cells to identify *TCF4*‐regulated genes. Our data shows that *TCF4* loss activates distinct but overlapping gene expression profiles in both cell lines (Table S1). Supporting the *TCF4* KD functional analysis described above, Gene Set Enrichment Analysis (GSEA) revealed negative enrichment for the hallmark gene sets involved in G2/M checkpoint, E2F targets, mitotic spindle, and MYC targets (FDR < 0.01, Fig. 4A,B).

Next, we examined the genome‐wide occupancy of TCF4 by chromatin immunoprecipitation (ChIP) followed by next‐generation sequencing (ChIP‐seq) analysis in Kelly and SK‐N‐AS cells. Using the Enrichr [45] database, we found enrichment for the genes of the proneuronal and MES subtypes among the TCF4 regulated targets. For instance, in Kelly cells, TCF4 controls factors that are downstream targets of MES factors (TWIST1, PRRX1, and SNAI2) and ADRN factor HAND1 (Fig. 4C). In SK‐N‐AS, *TCF4* regulates genes downstream of *EZH2, E2F2, MYCN*, and *HAND2* (Fig. 4D).

**Fig. 5:**
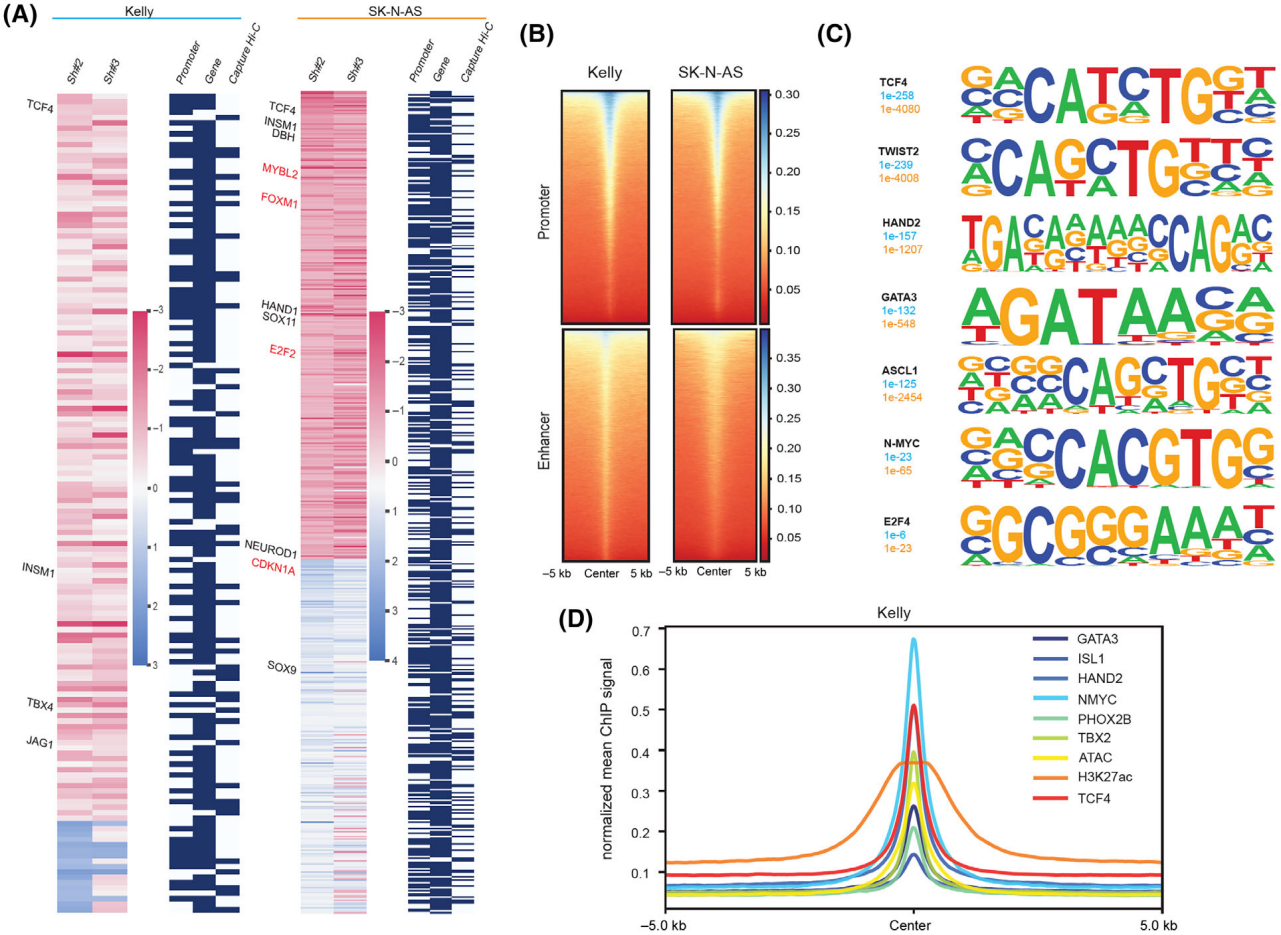
TCF4‐dependent regulatory network in neuroblastoma. (A) Heatmap image represents genes down‐ or up‐regulated in both Kelly and SK‐N‐AS cells after TCF4 knockdown using two different shRNAs (#2, #3) *n* = 3 biological replicates, containing a TCF4 ChIP‐Seq peak within the promoter (1000 bp from TSS based on gene orientation) (2 biological replicates), the gene body or capture Hi‐C data. Highlighted in red are DREAM complex components. (B) Heatmap indicating the binding intensity of TCF4 at promoters or enhancers (Homer annotation) within 5 kb of ChIP‐seq peaks in the Kelly and SK‐N‐AS cell lines. The color scale shows the intensity of the distribution signal. *n* = 2 biological replicates. (C) Enriched DNA‐binding motifs identified by HOMER corresponding to known transcription factors. (D) Aggregated ChIP‐seq signals for TCF4, H3K27ac, ATAC, MYCN, and the CRC members HAND2, PHOX2B, GATA3, ISL1, ASCL1 peaks in the Kelly cell line for the regions (−5000 to +5000 bp) from the TCF4 peak summits of all TCF4 peaks. Two replicates in Kelly are depicted.

The integration of ChIP‐seq and RNA‐seq data for *TCF4* KD revealed genes directly regulated by TCF4 in both NB cell lines (Fig. 5A, Table S2). Our integrative analysis of *TCF4* KD in SK‐N‐AS cells revealed that TCF4 contributes to NB oncogenesis and regulates sympathetic neurogenesis factors (HAND1, INSM1, NEUROD1, SOX11, DBH), and MES EMT markers such as SNAI2 (Fig. 5A, Table S2). TCF4 also regulates genes involved in driving proliferation, such as E2F‐FOXM1 core members, cell cycle regulatory DREAM complex genes, and genes of distinct oncogenic pathways required for NB proliferation (*CDKN1A, E2F1, E2F2, FOXM1, MYBL2*) (Fig. 5A). Moreover, we confirmed using (qRT‐PCR) that suppression of *TCF4* expression leads to a significant decrease in DREAM complex components expression (*E2F1, E2F2, FOXM1*, and *MYBL2*) (Fig. S5f). Another cell cycle regulator, EZH2, which is involved in epigenetic silencing of tumor‐suppressor genes by H3K27 methylation [46] was identified as a putative direct target of TCF4 (Table S2).

Next, using H3K27ac as a marker for active chromatin, we confirmed the enrichment of TCF4 binding to active promoter and enhancer regions (Fig. 5B). HOMER motif analysis shows that regions bound by TCF4 were also enriched for several CRC factors (Fig. 5C). We also observed enrichment of the E2F motif at the TCF4 binding sites in both cells (Fig. 5C). The GSEA combined with the ChIP‐seq data suggest that TCF4 regulates cell cycle progression through direct transcriptional regulation of E2F‐FOXM1 and their target genes. Moreover, we found that most of the NB‐specific regions of open chromatin overlapped with the TCF4 binding peaks in both cell lines (Fig. S5a–c). Finally, we explored the putative role of TCF4 in Kelly cells. Here, we integrated ChIP‐seq tracks for TCF4 with published data reported for ADRN CRC factors. As shown in (Fig. 5D) several factors, including MYCN and HAND2, were enriched around TCF4 binding sites. To further explore the relationship between TCF4 and MYCN in‐depth, we integrated previously published MYCN ChIP‐seq data [8, 38] with our TCF4 ChIP data. We observed a strong MYCN intensity signal at TCF4 binding sites (Fig. S5a). Many TCF4 binding sites are shared with MYCN, both at promoters and enhancers, which are sites of MYCN enhancer invasion and are associated with the MYCN oncogenic program (Fig. S5a). TCF4 peaks that overlap with MYCN peaks at SEs in both cell lines show enrichment for many CRC TFs, among which HAND2, TBX2, and MYCN are at the top of the list of enriched factors, as identified by ChIP Enrichment Analysis (ChEA) [45] (Fig. S5d,e). These data suggest collaborative roles among these factors and TCF4 in regulating NB gene expression programs and potentially physical interactions. However, using a published dataset of CRC TFs binding peaks in Kelly cells, we did not observe a strong overlap of TCF4 peak summits with the binding sites of these CRC factors at the *TCF4* locus (Fig. S5b). Moreover, in our RNA‐seq data, we did not observe a strong effect of *TCF4* KD on the expression of the known CRC members. This data suggests that *TCF4* is not a canonical CRC member but is regulated in parallel to these CRCs and facilitates their downstream effects. Hence, we propose that *TCF4* is a member of the extended regulatory network, reported as SE‐associated genes whose enhancers and promoters are bound by CRC TFs and work downstream of these factors to modulate their effect [7].

### 3.6 TCF4 interactome in NB cell lines

**Fig. 6:**
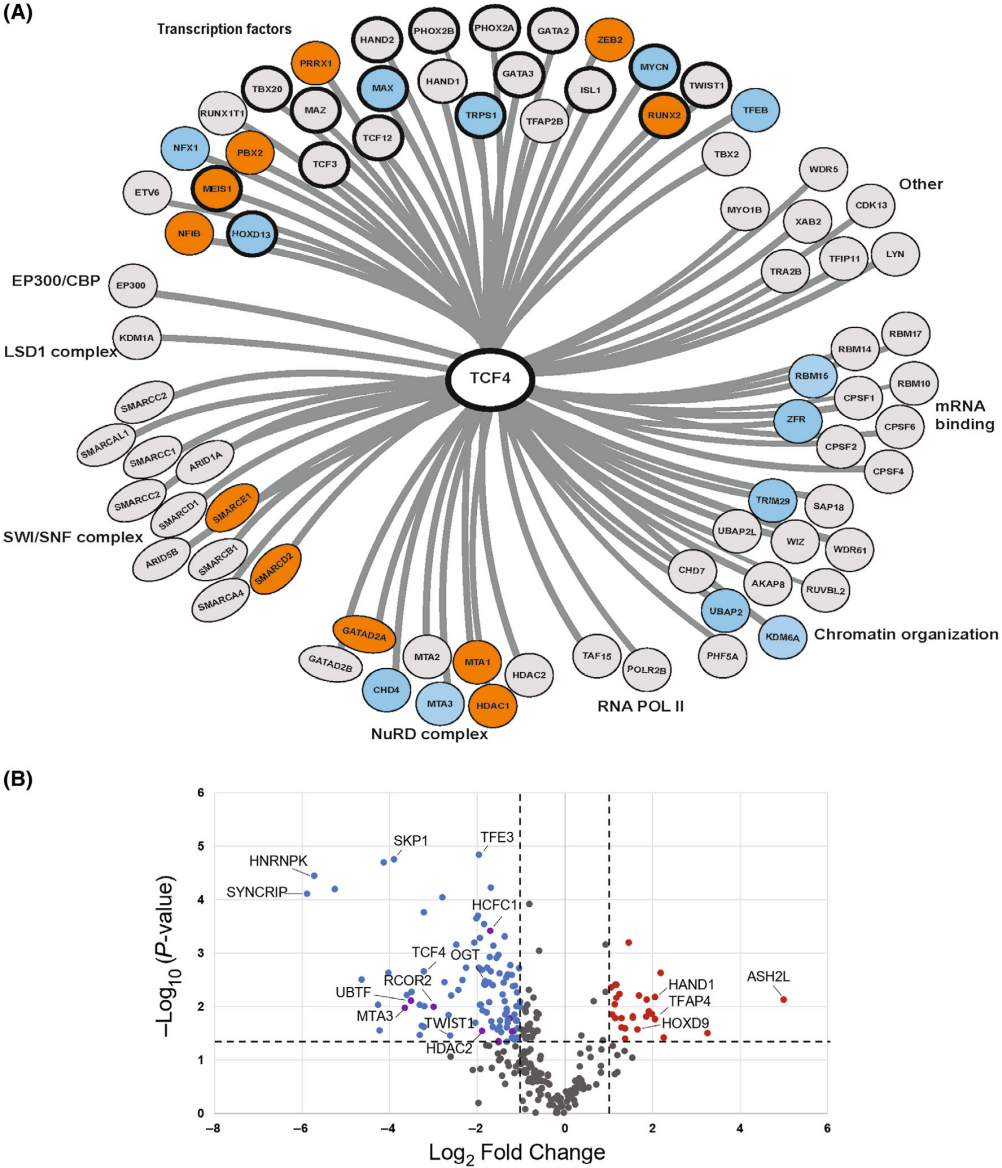
TCF4 interactome in neuroblastoma. (A) Proteins interacting with TCF4 in both Kelly and SK‐N‐AS neuroblastoma cells, identified by immunoprecipitation coupled to mass spectrometry (IP‐MS). Normal rabbit IgG was used as a negative control. Identified proteins are high‐confidence proteins identified in at least two independent (IP‐MS) reactions per cell line, found in both Kelly and SK‐N‐AS cells. *n* = 3 independent experiments. TCF4 interaction partners shared between Kelly and SK‐N‐AS cells are denoted in gray, TCF4 interactors identified in Kelly cells only are in blue, and SK‐N‐AS only are in orange. Bold circles represent transcription factors identified as TCF4 interactors in the (IP‐MS) analysis and have enriched DNA‐binding motifs identified in the TCF4 ChIP‐seq in both cell lines. (B) Volcano plot of HAND2 interacting proteins identified by IP‐MS with fold‐change versus significance of the change due to TCF4 silencing. Proteins that are significantly lost (blue or purple) or gained (red) after TCF4 silencing. *n* = 3 independent experiments. Purple points indicate proteins that complex with HDAC proteins. Dash lines demarcate proteins that are significantly changed (*P* < 0.05) and have a fold‐change greater than ±2‐fold.

Next, we performed TCF4 IP‐MS using total cellular extracts from NB cells to determine the TCF4 interactome in NB (Fig. S6d). Interacting proteins present in at least two purifications of the TCF4 protein were included (Table S3; see Section 2 for inclusion criteria). The TCF4 interaction network in NB compromises many ADRN and MES CRC TFs and multiple chromatin remodeling complexes, such as the nucleosome remodeling and deacetylase (NuRD) complex and the SWI‐SNF complex (Fig. 6A). Interestingly, there was a strong overlap between TFs identified as part of the TCF4 interactome in NB cell lines and motifs enriched in our TCF4 ChIP‐seq data (Fig. 6A).

To better define TCF4's contribution to transcriptional complexes, we performed IP‐MS studies of the core ADRN TF HAND2 identifying proteins that are lost after dox‐inducible shRNA silencing of TCF4 in Kelly cells (Fig. 6B and Table S4). The TCF4 sh#2 cell line was treated with dox to induce TCF4 silencing followed by IP‐MS using a HAND2 antibody. Our data indicate that TCF4 recruits heterogenous nuclear ribonucleoproteins (hnRNPs), such as SYNCRIP and HNRNPK [47]. SYNCRIP stabilizes c‐MYC mRNA [48] and regulates early neuronal differentiation through multiple mRNAs [49]. HNRNPK is also proposed to function as a TF that maintains stemness [50]. Additionally, TCF4 brings TFE3, which maintains neural stem‐progenitor cells and regulates neural differentiation, to HAND2 transcription factor [51]. TCF4 also recruits host cell factor 1 (HCFC1) that forms complexes that regulate cell cycle through interactions with MYC and E2Fs [52, 53]. Mutations that disrupt c‐MYC interactions with HCFC1 block c‐MYC transforming potential *in vitro* and *in vivo* indicating HCFC1 is essential for c‐MYC oncogenesis [54].

We find TCF4 recruits multiple factors (USP7, HDAC2 and SKP1) known to post‐translationally modify (PTM) MYC oncoproteins to increase their activity and stability [55, 56] (Fig. 6B). USP7 is a unique deubiquitinase that stabilize c‐MYC and N‐MYC by counteracting polyubiquitination dependent proteosome degradation [57, 58, 59]. Histone deacetylase 2 (HDAC2) and subunits of HDAC complexes, such as RCOR2 of CoREST, a chromatin regulatory complex, and MTA3 of the nucleosome remodeling and deacetylase NuRD complex are also recruited (Fig. 6B). TCF4 also recruits SKP1 of the SKP1‐Cullin‐F‐Box (SCF) E3‐ubiquitin ligase complex. HDAC2 is a coactivator of c‐MYC in medulloblastoma by facilitating transcription factor licensing of c‐MYC [60]. Transcription factor licensing requires mono‐ubiquitination of c‐MYC by E3‐ubiquitin ligases, like SCF, which improves the binding of MYC oncoproteins to weak E‐box DNA consensus sequences [60, 61]. HDAC2 can facilitate transcription factor licensing by removing lysine acetylation that block the ubiquitination necessary for the transcription factor licensing of MYC [60, 61]. These data reveal a model for TCF4, which colocalizes in the genome with HAND2 and MYCN, for recruiting multiple factors to HAND2 to promote MYC function and stability, E2F activity and MYC transforming ability.

## 4 Discussion

Neuroblastoma tumors display startling heterogeneity, compromising the populations of both ADRN and MES lineage [10]. This heterogeneity provides NB with high transcriptional plasticity, allowing tumors to escape therapeutic treatment or relapse [5, 9, 62]. NB identity is determined by lineage‐specific transcription factors driven by cell type‐specific SEs [5]. In this study, we identify TCF4 as a critical NB dependency gene that is shared across different NB lineage states. Our functional analysis demonstrated that loss of TCF4 dramatically decreased cell proliferation, induced apoptosis, and inhibited tumor growth *in vivo*.

We found that *TCF4* silencing in NB cells downregulated the expression of *E2F* genes, *FOXM1* and *MYBL2. E2Fs, FOXM1, and MYBL2*, as part of the DREAM complex, coordinately regulate multiple stages of cell cycle progression, including G2M transition (Fig. 5A and Fig. S5f) [63]. MYBL2 and FOXM1 are important oncogenic factors, and MYBL2 is known to regulate MYCN in NB [64]. Furthermore, a recent study identified TBX2 as a member of the ADRN CRC family that similarly regulates E2Fs, MYBL2, and FOXM1 [65]. Our results with recent findings for TBX2, HAND2 and TWIST1 propose a role for TCF4 in regulating FOXM1/E2F‐driven gene regulatory networks that control cell cycle progression in cooperation with MYCN, TBX2, and the TCF4 dimerization partners HAND2 and TWIST1.

To functionally determine how TCF4 regulates these oncogenic programs, we integrated our TCF4 ChIP‐seq data with publicly available data for CRC ADRN factors in Kelly cells and found a high concordance of DNA occupancy by TCF4 with HAND2, MYCN, and TBX2 proteins. To test whether TCF4 and CRC TFs physically interact, we performed IP‐MS analysis and identified TCF4 protein–protein interactions in two NB cell lines. The analysis revealed enrichment in the network of multiple ADRN and MES NB CRC factors. MYC oncoproteins drive high‐risk NB and by additional IP‐MS analysis, we determined that TCF4 recruits to HAND2 multiple factors that are supportive of MYC and E2F function. This includes factors important for augmenting transcription and mRNA processing of MYC targets to lineage specifying class II bHLH TFs.

N‐MYC typically binds canonical E‐boxes at promoters, but at oncogenic levels of expression *N‐MYC* invades and binds to weak E‐boxes at the enhancers of genes associated with NB aggressiveness [14] which is also observed with c‐Myc in other cancers [17]. Interestingly, TCF4 recruits HDAC2 and SKP1 that are known to facilitate transcription factor licensing of MYC [60, 66, 67] which could explain recent work establishing that TCF4 heterodimerization partners HAND2 and TWIST1 increase N‐MYC activity at SEs with weak E‐box consensus sites. Our data indicates that *TCF4* is a shared collaborative factor with TWIST1 or HAND2 involved in facilitating TF‐DNA binding of MYCN, which we propose is through transcription factor licensing, to regulate downstream oncogenic programs. Though class I bHLH TFs (TCF3, TCF4, and TCF12) are important for cancer, development, and immunity, and associated with neural disorders, how they functionally contribute to these processes is poorly understood [21, 68, 69]. Our data identified the importance of TCF4, and potentially other class I bHLH TFs, in bridging lineage specifying TFs to MYC proteins through their support of MYC transcriptional amplification of class II bHLH TF specified developmental programs.

## 5 Conclusion

Taken together, we show for the first time that TCF4 is a critical NB dependency gene that significantly contributes to NB identity states. Additionally, our data suggest a role for TCF4 in regulating (FOXM1)/E2F‐driven gene regulatory networks that control cell cycle progression in cooperation with MYCN, TBX2, and the TCF4 dimerization partners HAND2 and TWIST1. Here, we propose a model that posits that TCF4 acts through recruiting multiple factors known to regulate MYC function to sites of colocalization between critical NB transcription factors, TCF4 and MYC oncoproteins.

## Conflict of interest

The authors declare no conflict of interest.

## Author contributions

KWF and NAA were involved in conception and design, study supervision, writing of the manuscript, and development of methodology. KWF, NAA, and MW were involved in experimental design. NAA, CD, SA, and RRO were involved in performing experiments. KWF, NAA, DS, and YC were involved in analysis and interpretation of data (e.g., statistical analysis, sequencing data, and computational analysis). KWF, NAA, MW, RRO, DS, and YC were involved in review and/or revision of the manuscript. NAA and DS were involved in administrative and technical support (reporting or organizing data and constructing databases).

### Peer review

The peer review history for this article is available at https://www.webofscience.com/api/gateway/wos/peer‐review/10.1002/1878‐0261.13714.

## Supporting information


**Fig. S1.** TCF4 is a shared factor across ADRN and MES NB cell lines.
**Fig. S2.** Knockdown of TCF4 dramatically decreases cell proliferation and induces apoptosis.
**Fig. S3.** TCF4 loss dramatically decreases cell proliferation in NB cell lines.
**Fig. S4.** TCF4 knockdown induces apoptosis in NB cell lines.
**Fig. S5.** TCF4 shows a high concordance of DNA occupancy with CRC proteins.
**Fig. S6.** The full blots, where portions of blots have been presented in the main paper.

**Table S1.** Differential gene expression after knocking down TCF4 in Kelly and SK‐N‐AS cell lines using two different shRNAs (#2, #3).

**Table S2.** TCF4 target genes in both Kelly and SK‐N‐AS based on TCF4 ChIP Seq and Promoter Capture Hi‐C.

**Table S3.** TCF4 interactors in Kelly and SK‐N‐AS NB cell lines.

**Table S4.** IP/MS HAND2 interactors following TCF4 knockdown in Kelly cells.

## Data Availability

RNA, ChIP, and ATAC sequencing data are available online through the Gene Expression Omnibus (GEO) portal. The accession numbers for this SuperSeries were GSE222212, GSE222212, and GSE222214.
