# Supplementary material for: Transcription factor 4 is a key mediator of oncogenesis in neuroblastoma by promoting MYC activity
Source: Mol Oncol. 2024 Aug 9;19(3):808–24. doi: 10.1002/1878-0261.13714 (PMC11887674; doi:10.1002/1878-0261.13714)
Supplement: Supplementary file 1 — Fig. S1. TCF4 is a shared factor across ADRN and MES NB cell lines. Fig. S2. Knockdown of TCF4 dramatically decreases cell proliferation and induces apoptosis. Fig. S3. TCF4 loss dramatically decreases cell proliferation in NB cell lines. Fig. S4. TCF4 knockdown induces apoptosis in NB cell lines. Fig. S5. TCF4 shows a high concordance of DNA occupancy with CRC proteins. Fig. S6. The full blots, where portions of blots have been presented in the main paper. [file MOL2-19-808-s004.zip › SupplementaryFigurelegends.docx]

**Supplementary Figure 1. TCF4 is a shared factor across adrenergic and Mesenchymal neuroblastoma cell lines. a** Dose-response curve of cells 4 days following treatment with half-log dilutions of JQ1. Cell lines include Kelly, IMR32 (adrenergic), SK-N-AS and mouse NCCs (Mesenchymal/STEM). Results were normalized to control ± S.E. n = 3 independent experiments. **b** STRING database analysis demonstrates TCF4 putative protein-protein interactions. Red nodes indicate known MES transcription factors (TFs) and blue nodes indicate known adrenergic transcription factors. **c** A relationship plot generated from the Super-enhancer database (SEdb 2.0) using the SE-based TF-GENE Analysis program to comprehensively analyze TFs gene pairs mediated by super-enhancers in NB cell lines.

**Supplementary Figure 2. Knockdown of TCF4 dramatically decreases cell proliferation and induces apoptosis. a** CyQuant proliferation assay performed using Kelly TCF4 sh #1, #2, #3 cell lines compared to empty vector control (NTC) cell line 5 days after doxycycline treatment. Results were normalized to control + S.E. n = 3 independent experiments. (*p<0.05**p<0.01, ***p<0.001 vs. control). **b** Colony formation assays were performed following TCF4 knockdown in Kelly cells. Cells were cultured for 10 days with or without 1 μg/mL of doxycycline. **c** % of cells in each phase of the cycle 5 days following TCF4 knockdown in the Kelly cell line. Cell cycle was assayed by flow cytometry. (*p<0.05**p<0.01, ***p<0.001 vs. control). **d** Quantitative analysis of the percentage of apoptotic cells (Annexin V + /FITC +) in Kelly TCF4 stable cell lines treated with or without 1 μg/mL doxycycline for 5 days. (*p<0.05**p<0.01, ***p<0.001 vs. control). **e** Western blot of cleaved PARP protein levels in Kelly and SK-N-AS cells 5 days after doxycycline treatment. Data are presented as the mean ± S.E. **f** TCF4 mRNA level determined using real-time PCR following TCF4 overexpression in SK-N-AS cells. These cells were transfected with TCF4 cDNA vector. Three resistant clones stably overexpressing TCF4 were culture and expanded. Results were normalized to parental SK-N-AS cell line + S.E.

**Supplementary Figure 3. TCF4 loss dramatically decreases cell proliferation in neuroblastoma cell lines.** Raw data for cell cycle analysis of (**a)** Kelly and (**b**) SK-N-AS stable NB cells after doxycycline exposure. Cell cycle distribution was analyzed by flow cytometry. G1, S, and G2/M show the cell cycle phase. Data from one of three experiments are shown, including flow cytometry plot and summarized data.

**Supplementary Figure 4. TCF4 knockdown induces apoptosis in neuroblastoma cell lines.** Apoptosis detection using Annexin-V FITC/PI staining. a Flow cytometry data indicated an elevated level of apoptosis was observed in the two NB stable cells line used; Kelly and SK-N-AS after doxycycline treatment compared to NTC control. Data from one of three experiments are shown, including flow cytometry plot and summarized data.

**Supplementary Figure 5. TCF4 shows a high concordance of DNA occupancy with core regulatory circuitry proteins. a** ChIP fragment depth for TCF4, H3K27ac, ATAC, MYCN, and the core regulatory circuitry (CRC) members HAND2, PHOX2B, GATA3, ISL1, ASCL1 peaks in the Kelly cell line for the regions (+/−5.0 Kb) from the TCF4 peak summits of all TCF4 peaks (left), peaks located at promotor (center), and peaks located at enhancers (right). **b** ChIP-seq gene tracks showing the binding locations of adrenergic CRC members at the TCF4 gene locus in Kelly cells. Also shown are the enhancer marker (H3K27ac) and open chromatin (ATAC-seq) at the TCF4 gene locus in Kelly cells. **c** ChIP-seq gene tracks showing the enhancer marker (H3K27ac) and open chromatin (ATAC-seq) at the TCF4 gene locus in SK-N-AS cells. **d,e** Enrichr pathway analysis of TCF4 peaks that overlap with MYCN peaks at SEs in Kelly and SK-N-AS cells using the ChIP Enrichment Analysis (ChEA). The lists of genes were analyzed based on the combined score ranking. p-value < 0.05 was used as the significance threshold. **f** Quantitative RT-PCR analysis showing of DREAM complex components (E2F1, E2F2, FOXM1, and MYBL2) after TCF4 knockdown in Kelly and SK-N-AS stable cells. Results were normalized to control + S.E. n = 3 independent experiments. (*p<0.05**p<0.01, ***p<0.001 vs. control).

**Supplementary Figure 6. The full blots, where portions of blots have been presented in the main paper. a** For figure 1e**. b** For figure 2b**. c** For figure 3e**. d** For figure 6 a and b**. e** For Supplementary Figure 2.
