# Supplementary figures and images for: Transcription factor 4 is a key mediator of oncogenesis in neuroblastoma by promoting MYC activity
Source: Mol Oncol. 2024 Aug 9;19(3):808–24. doi: 10.1002/1878-0261.13714 (PMC11887674; doi:10.1002/1878-0261.13714)

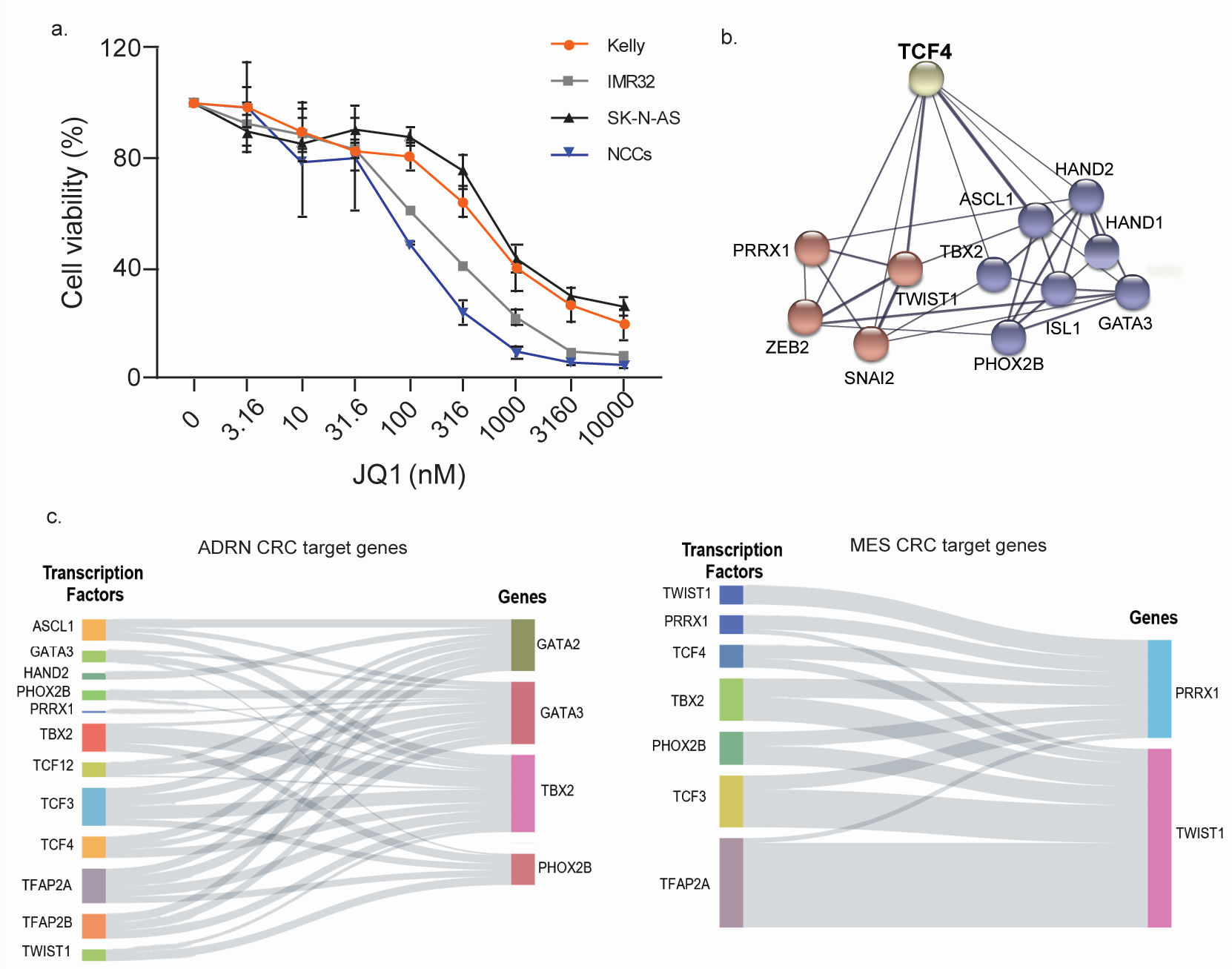

Supplement: Supplementary file 1 — Fig. S1. TCF4 is a shared factor across ADRN and MES NB cell lines. Fig. S2. Knockdown of TCF4 dramatically decreases cell proliferation and induces apoptosis. Fig. S3. TCF4 loss dramatically decreases cell proliferation in NB cell lines. Fig. S4. TCF4 knockdown induces apoptosis in NB cell lines. Fig. S5. TCF4 shows a high concordance of DNA occupancy with CRC proteins. Fig. S6. The full blots, where portions of blots have been presented in the main paper. [file MOL2-19-808-s004.zip › mol213714-sup-0001-Figure_S1.tif]

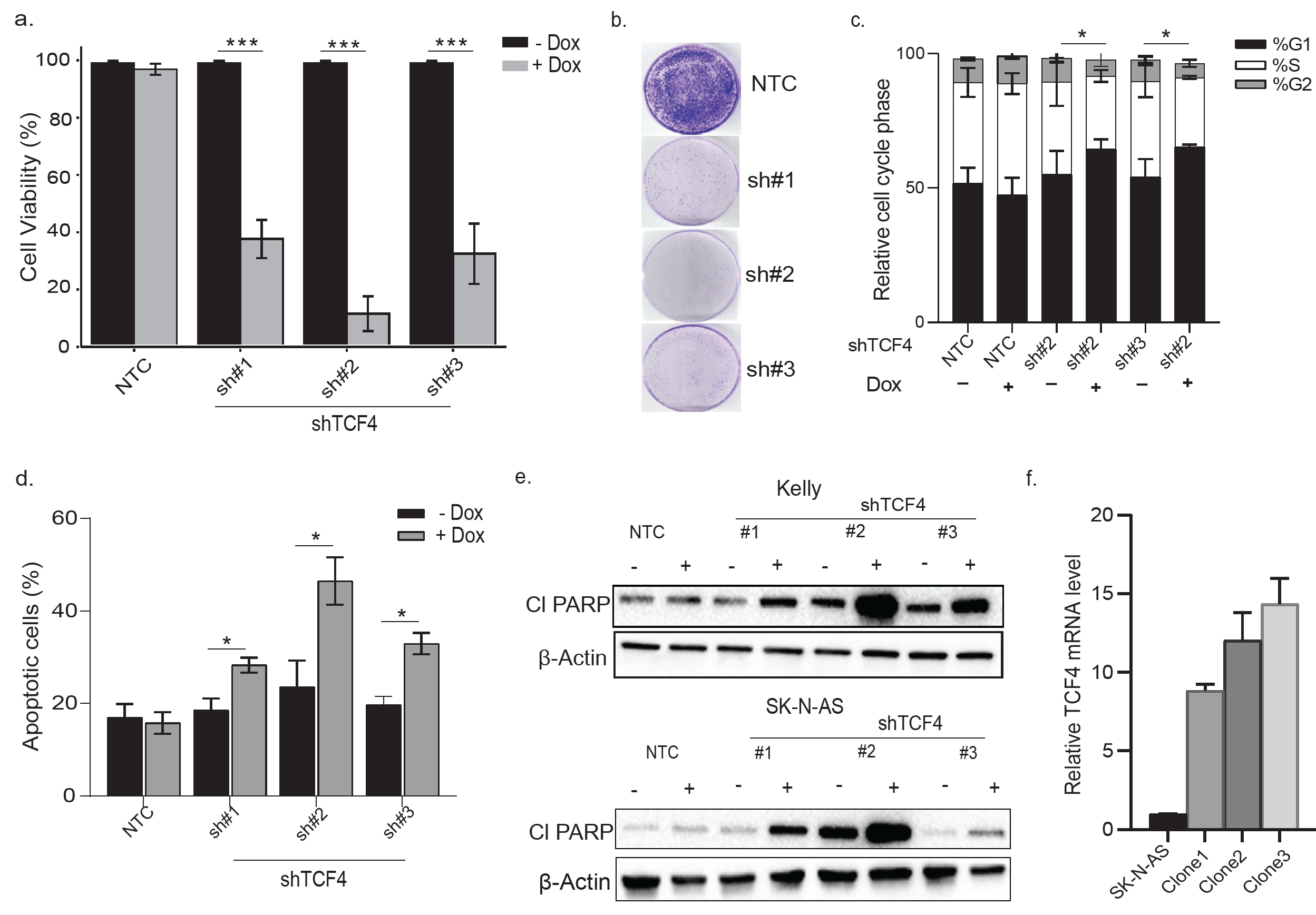

Supplement: Supplementary file 1 — Fig. S1. TCF4 is a shared factor across ADRN and MES NB cell lines. Fig. S2. Knockdown of TCF4 dramatically decreases cell proliferation and induces apoptosis. Fig. S3. TCF4 loss dramatically decreases cell proliferation in NB cell lines. Fig. S4. TCF4 knockdown induces apoptosis in NB cell lines. Fig. S5. TCF4 shows a high concordance of DNA occupancy with CRC proteins. Fig. S6. The full blots, where portions of blots have been presented in the main paper. [file MOL2-19-808-s004.zip › mol213714-sup-0002-Figure_S2.tif]

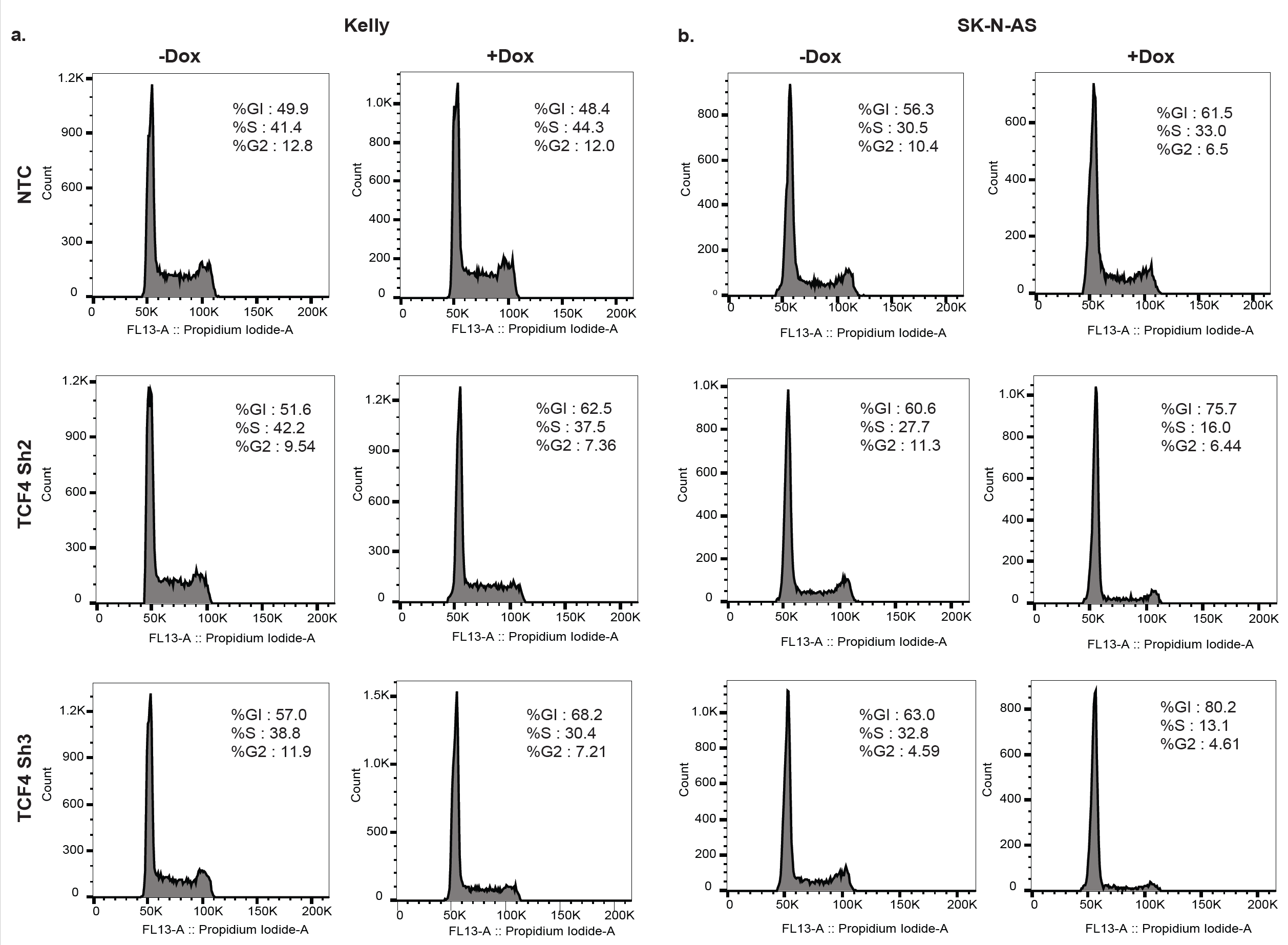

Supplement: Supplementary file 1 — Fig. S1. TCF4 is a shared factor across ADRN and MES NB cell lines. Fig. S2. Knockdown of TCF4 dramatically decreases cell proliferation and induces apoptosis. Fig. S3. TCF4 loss dramatically decreases cell proliferation in NB cell lines. Fig. S4. TCF4 knockdown induces apoptosis in NB cell lines. Fig. S5. TCF4 shows a high concordance of DNA occupancy with CRC proteins. Fig. S6. The full blots, where portions of blots have been presented in the main paper. [file MOL2-19-808-s004.zip › mol213714-sup-0003-Figure_S3.tif]

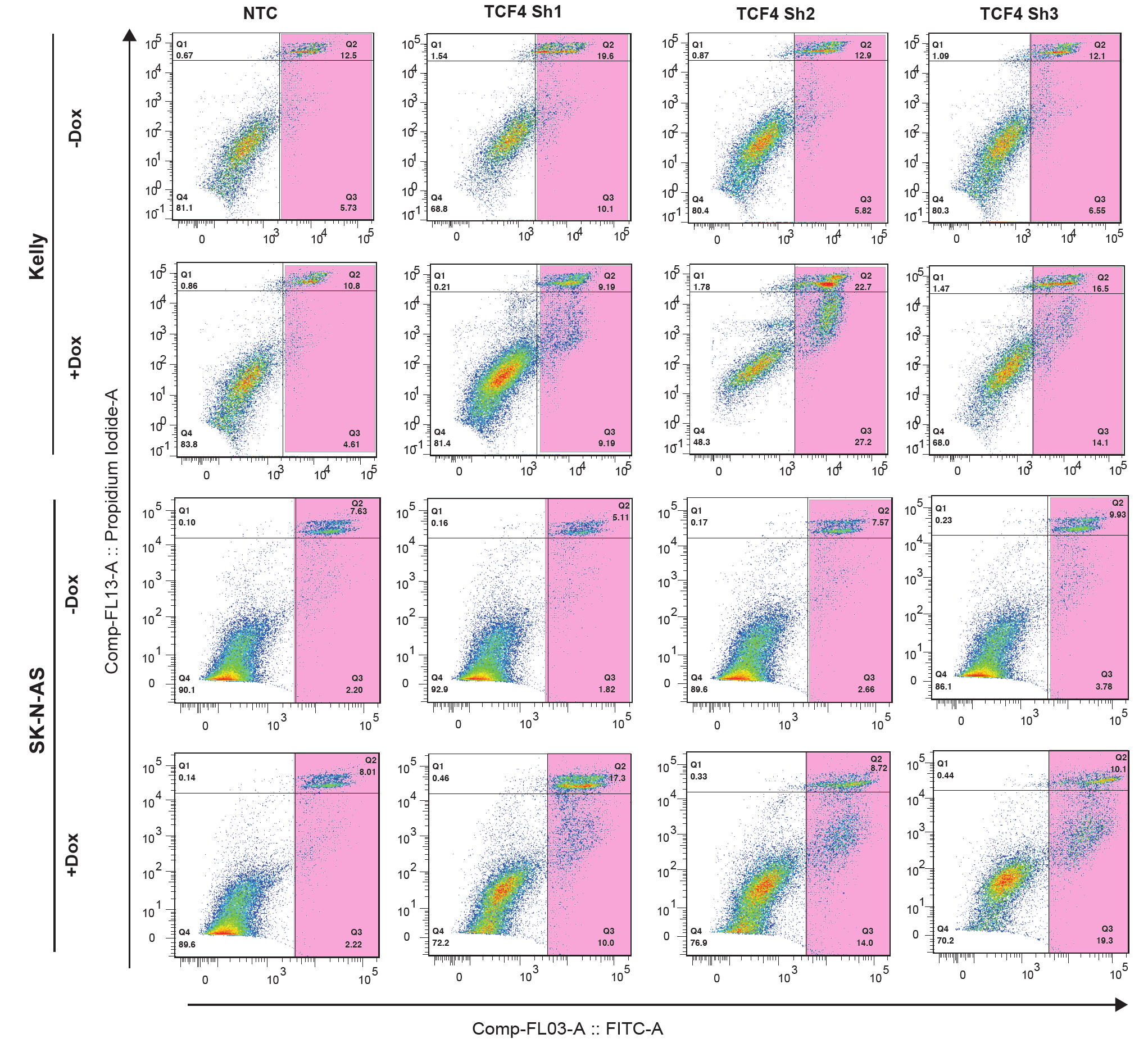

Supplement: Supplementary file 1 — Fig. S1. TCF4 is a shared factor across ADRN and MES NB cell lines. Fig. S2. Knockdown of TCF4 dramatically decreases cell proliferation and induces apoptosis. Fig. S3. TCF4 loss dramatically decreases cell proliferation in NB cell lines. Fig. S4. TCF4 knockdown induces apoptosis in NB cell lines. Fig. S5. TCF4 shows a high concordance of DNA occupancy with CRC proteins. Fig. S6. The full blots, where portions of blots have been presented in the main paper. [file MOL2-19-808-s004.zip › mol213714-sup-0004-Figure_S4.tif]

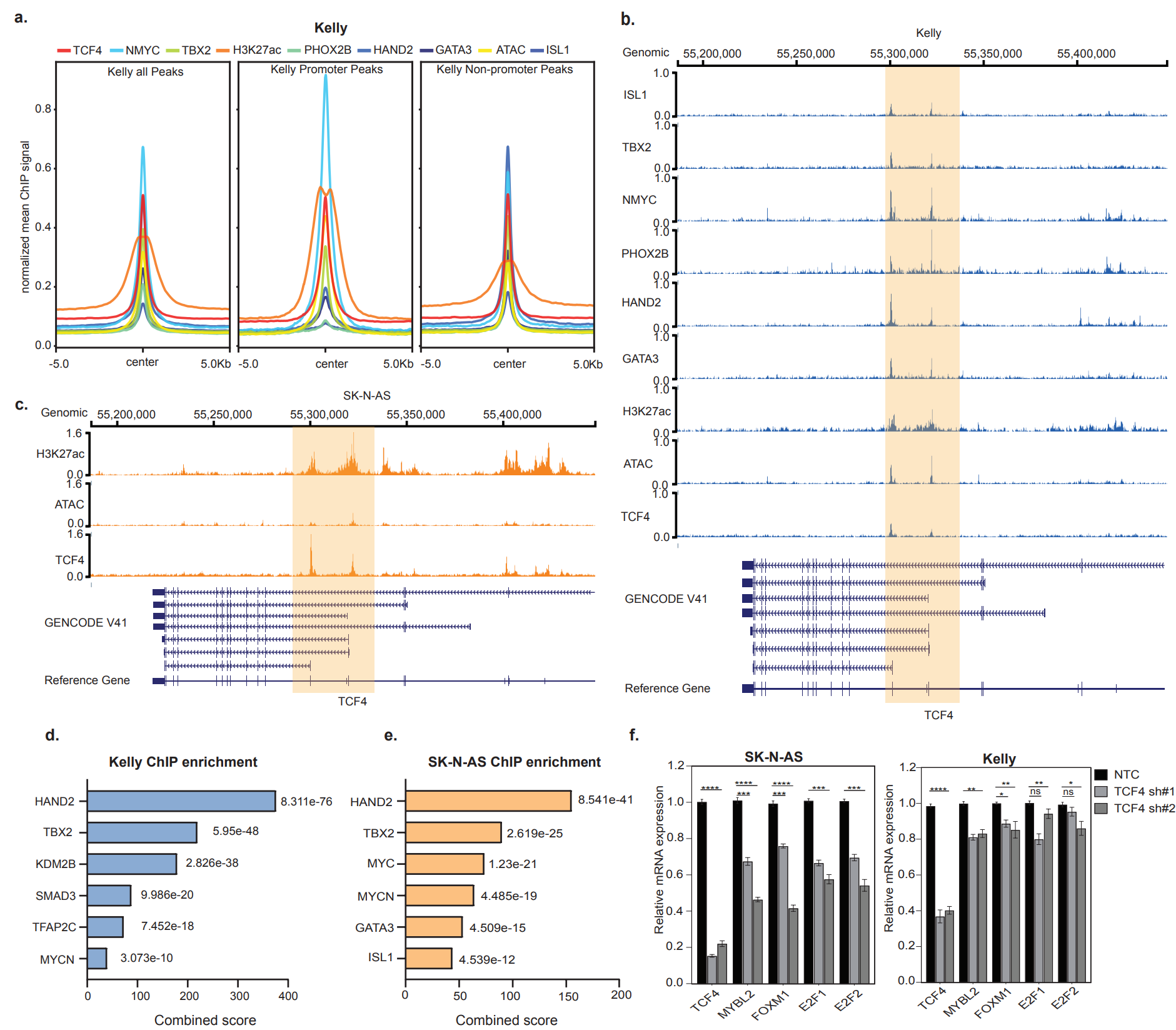

Supplement: Supplementary file 1 — Fig. S1. TCF4 is a shared factor across ADRN and MES NB cell lines. Fig. S2. Knockdown of TCF4 dramatically decreases cell proliferation and induces apoptosis. Fig. S3. TCF4 loss dramatically decreases cell proliferation in NB cell lines. Fig. S4. TCF4 knockdown induces apoptosis in NB cell lines. Fig. S5. TCF4 shows a high concordance of DNA occupancy with CRC proteins. Fig. S6. The full blots, where portions of blots have been presented in the main paper. [file MOL2-19-808-s004.zip › mol213714-sup-0005-Figure_S5.tif]

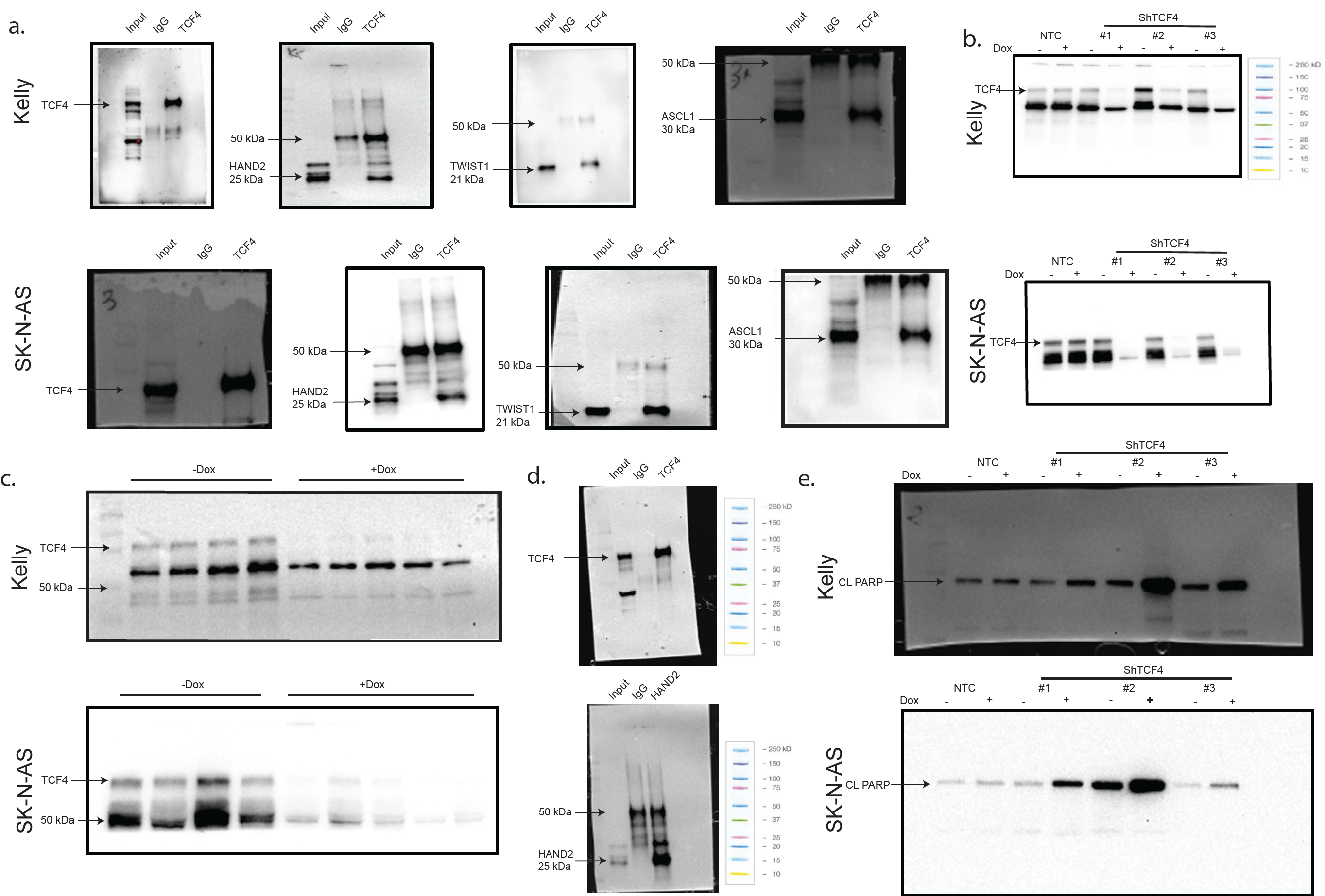

Supplement: Supplementary file 1 — Fig. S1. TCF4 is a shared factor across ADRN and MES NB cell lines. Fig. S2. Knockdown of TCF4 dramatically decreases cell proliferation and induces apoptosis. Fig. S3. TCF4 loss dramatically decreases cell proliferation in NB cell lines. Fig. S4. TCF4 knockdown induces apoptosis in NB cell lines. Fig. S5. TCF4 shows a high concordance of DNA occupancy with CRC proteins. Fig. S6. The full blots, where portions of blots have been presented in the main paper. [file MOL2-19-808-s004.zip › mol213714-sup-0006-Figure_S6.tif]
